# Glucose-derived carbon materials with tailored properties as electrocatalysts for the oxygen reduction reaction

**DOI:** 10.3762/bjnano.10.109

**Published:** 2019-05-21

**Authors:** Rafael Gomes Morais, Natalia Rey-Raap, José Luís Figueiredo, Manuel Fernando Ribeiro Pereira

**Affiliations:** 1 Associate Laboratory LSRE-LCM, Departamento de Engenharia Química, Faculdade de Engenharia, Universidade do Porto, R. Dr. Roberto Frias s/n, 4200-465 Porto, Portugal

**Keywords:** electrocatalysts, microporosity, nitrogen-doped carbon materials, oxygen reduction reaction, surface chemistry

## Abstract

Nitrogen-doped biomass-derived carbon materials were prepared by hydrothermal carbonization of glucose, and their textural and chemical properties were subsequently tailored to achieve materials with enhanced electrochemical performance towards the oxygen reduction reaction. Carbonization and physical activation were applied to modify the textural properties, while nitrogen functionalities were incorporated via different N-doping methodologies (ball milling and conventional methods) using melamine. A direct relationship between the microporosity of the activated carbons and the limiting current density was found, with the increase of microporosity leading to interesting improvements of the limiting current density. Regardless of the doping method used, similar amounts of nitrogen were incorporated into the carbon structures. However, significant differences were observed in the nitrogen functionalities according to the doping method applied: ball milling appeared to originate preferentially quaternary and oxidized nitrogen groups, while the formation of pyridinic and pyrrolic groups was favoured by conventional doping. The onset potential was improved and the two-electron mechanism of the original activated sample was shifted closer to a four-electron pathway due to the presence of nitrogen. Interestingly, the high pyridinic content related to a high ratio of pyridinic/quaternary nitrogen results in an increase of the onset potential, while a decrease in the quaternary/pyrrolic nitrogen ratio favors an increase in the number of electrons. Accordingly, the electrocatalyst with the highest performance was obtained from the activated sample doped with nitrogen by the conventional method, which combined the most appropriate textural and chemical properties: high microporosity and adequate proportion of the nitrogen functionalities.

## Introduction

Due to the recent increase in interest for more sustainable, renewable and cheaper energy, multiple conversion devices are being developed using new and innovative materials. Fuel cells are outstanding conversion devices, as they convert chemical energy directly into electrical energy with high efficiency and low emission of pollutants (the by products are water and heat) [1–2]. Fuel cells offer the best advantages for use with engines, as they are able to function as long as there is fuel, and for batteries, as they have similar characteristics under load conditions [1]. The performance of a fuel cell is mainly controlled by the oxygen reduction reaction (ORR) that takes place at the cathode [2], specifically by the electrocatalyst used for the reaction. The most commonly used electrocatalyst to supply faster kinetics and a four-electron pathway are platinum-based materials [3–4], which are costly and may assume up to 50% of the total cost of a fuel cell [5].

Transition metals [6–7], metal oxides [8–9] and carbon materials [3–4] have been widely studied as electrocatalysts in ORR due to their attractive physical and electrochemical properties. Among these materials, metal-free carbon materials have received tremendous attention due to their versatility and lower price in comparison with metal-based materials [2]. The main advantage of carbon materials is the possibility of modifying their physical and chemical properties resulting in a more electroactive material [2–3,10], which is an especially important feature for the ORR. The incorporation of heteroatoms like nitrogen [11–12], oxygen [13], sulfur [14–15], phosphorous [16–17] and boron [18–19] has been proven to enhance the electroactivity of carbon materials. Nitrogen has been the most studied heteroatom in the context of the reaction mechanism, since nitrogen-doped materials can achieve a four-electron pathway towards ORR [20–22]. However, there is some controversy related to the effect of the different nitrogen functionalities in the process. Some investigations suggest that the reduction of oxygen is promoted by pyridinic groups [23], while other researchers reported that quaternary nitrogen groups are the most active sites [24–25], and some studies assume that both functionalities contribute to enhancing the performance of the materials towards ORR [20]. In addition, a recent study with carbon nanotubes (CNTs) reported that an increase of the pyridinic-N/quaternary-N and pyridinic-N/pyrrolic-N ratios increases the electroactivity and that decreasing the quaternary-N/pyrrolic-N ratio increases the number of electrons involved in the ORR [12]. The electron density movement due to the presence of quaternary nitrogen favors the O_2_ dissociation, while the pyridinic nitrogen favors the bonding of oxygen to the neighboring carbon. Accordingly, the appropriate ratio between both nitrogen functionalities seems to be essential to improve the electroactivity of carbon materials. The differences reported on the influence of the nitrogen functionalities may be related to the nature and type of the carbon material employed, which in turn, depends on the precursors used and the method of synthesis applied.

Nitrogen-doped carbon materials have been synthesized by applying different doping methods to different types of materials, such as CNTs [12,23,26], graphene [20,25,27], carbon aerogels [15,28], carbon nanofibers [29], carbon nitrides [30], activated carbons [31] or mesoporous carbons [32–33]. Some of these materials are obtained from chemical compounds, fossil fuels or by complex and expensive synthesis procedures. In order to keep fuel cells as ecologically friendly as possible, the use of biomass as a carbon source appears to be an attractive alternative. In this context, hydrothermal carbonization (HTC) has appeared in recent years as an interesting strategy to obtain biomass-derived carbons due to its low cost and mild synthesis conditions, making the process environmentally friendly [34]. However, the main drawback of HTC is that the as-prepared hydrothermal carbon materials usually exhibit limited porosity and inadequate chemical properties for the ORR. To solve this problem, different strategies can be addressed: i) carbonization and activation methods to tailor the porosity and ii) the incorporation of heteroatoms to modify the surface chemistry, specifically by adding nitrogen functionalities. Biomass-derived carbons have been functionalized by applying thermal treatments in the presence of a gaseous or solid nitrogen precursor [35–36] or without any precursor in the case where the biomass already contains nitrogen in its constitution [11,37], and by in situ methods in which nitrogen precursors are introduced during the hydrothermal carbonization [38]. An additional strategy that can be applied to biomass processing is ball milling, which has been proposed as a green, cheap and easy method to incorporate nitrogen atoms and to modify the surface chemistry of carbon materials [39–40]. Some of these doping techniques have shown to provide materials with similar ORR performance as commercially available Pt/C electrocatalysts [39]. However, most of these studies have been focused only on the effect of nitrogen functionalities on the ORR, leaving aside the effect of porosity. In fact, although some studies suggest the importance of microporosity on the ORR [41], there is a lack of knowledge about its real effect on the ORR performance of nitrogen-doped porous carbon materials, and more specifically, of biomass-derived carbons.

Therefore, this study aims to prepare glucose-derived carbon materials with different textural and chemical properties and to correlate these properties with the performance towards ORR. Activated carbons with different microporosity were prepared by activating the samples at different times to determine the relationship between the textural properties and the ORR performance. Moreover, different doping strategies were applied to assess the effect of such methods on the incorporation of nitrogen functionalities and to evaluate the influence of the different functional groups on the response of the electrocatalyst towards the oxygen reduction reaction.

## Results and Discussion

### Effect of microporosity

**Figure 1:**
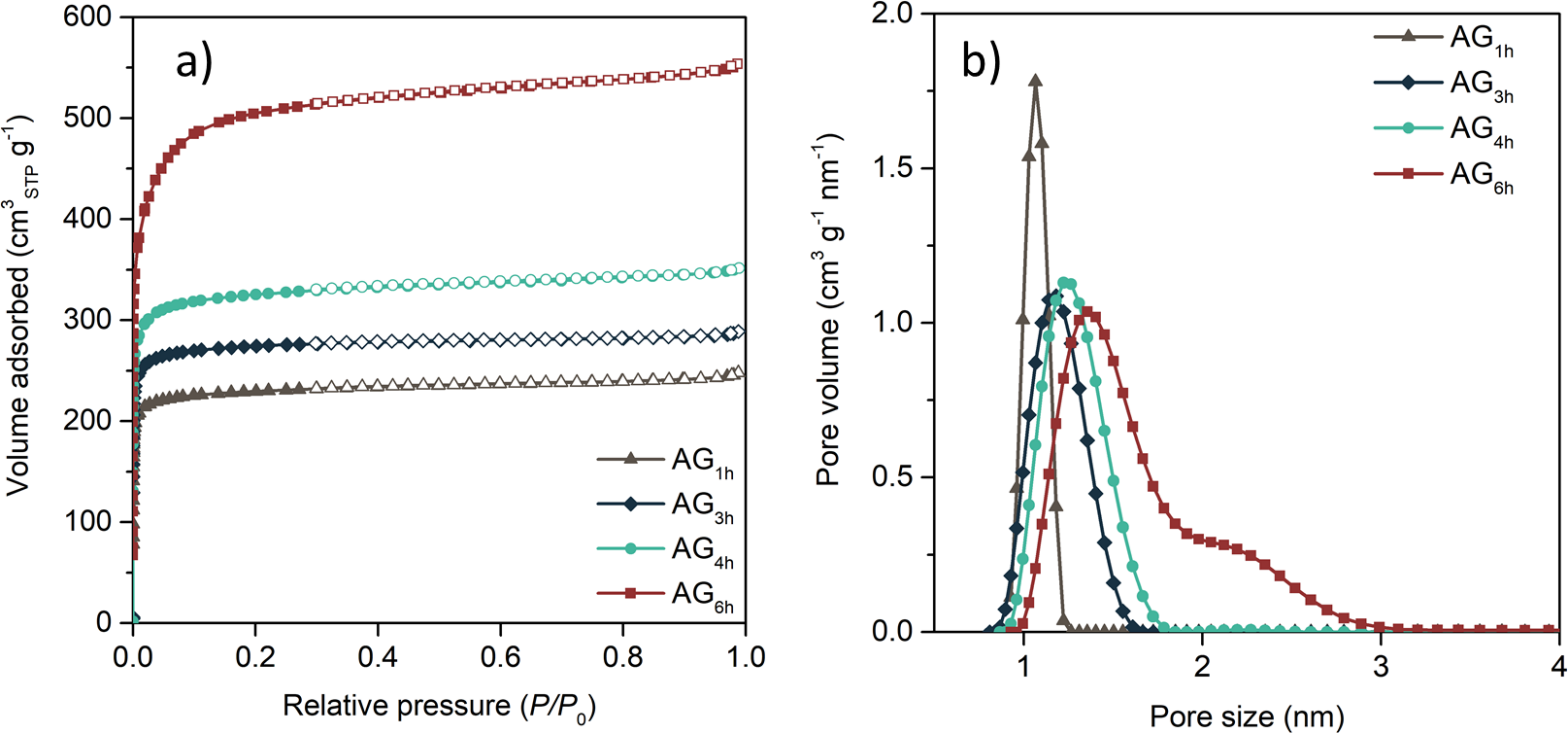
N_2_ adsorption/desorption isotherms (a) and pore size distributions (b) of the activated carbons.

The nitrogen adsorption–desorption isotherms and the pore size distributions obtained by applying the quenched solid density functional theory (QSDFT) are presented in Figure 1a and 1b, respectively.

Activated carbons display isotherms of type I according to the International Union of Pure and Applied Chemistry (IUPAC) classification, attributed to microporous materials. As expected, the volume of nitrogen adsorbed at low relative pressure increases with the time of activation, resulting in materials with a larger volume of micropores (Supporting Information File 1, Table S1). This effect is due to the reverse Boudouard reaction, which extracts carbon atoms from the carbon structure, developing the porosity of the material [42]. Accordingly, a prolonged contact time between the carbon material and the activating agent results in materials with a more developed microporosity. In addition, the pore size of the samples is also broadened by increasing the contact time (Figure 1b). The reaction occurring during the physical activation of the samples removes carbon atoms, giving rise to larger voids inside the particles, and hence, to larger pore sizes.

Regarding the chemical composition, the activated samples are mainly composed of carbon, with a smaller percentage of oxygen in the range of 2–4 wt % (Supporting Information File 1, Table S2). Although these values are not too high, oxygen functionalities can modify the electroactivity of carbon materials [13], therefore the nature of these functional group has been analyzed by temperature programmed desorption (TPD) experiments to evaluate possible differences in the oxygen functionalities. The total amount of CO (anhydrides, phenols, carbonyls) and CO_2_ (carboxylic acids, anhydrides, lactones) released was calculated from the corresponding TPD profiles, and the values obtained are compiled in Supporting Information File 1, Table S3. The CO_2_ and CO desorption profiles, obtained for samples AG_1h_ and AG_6h_ are also shown in Supporting Information File 1, Figure S1. The TPD profiles show that the sample with the highest degree of activation (AG_6h_) has a lower amount of phenols due to the longer time used for the activation. However, in general terms, the released CO and CO_2_ and their ratio are quite similar, so it can be assumed that there are no significant differences between the chemical composition of the samples, and therefore, any difference in the ORR performance can be exclusively related to the microporosity and/or the degree of activation.

**Figure 2:**
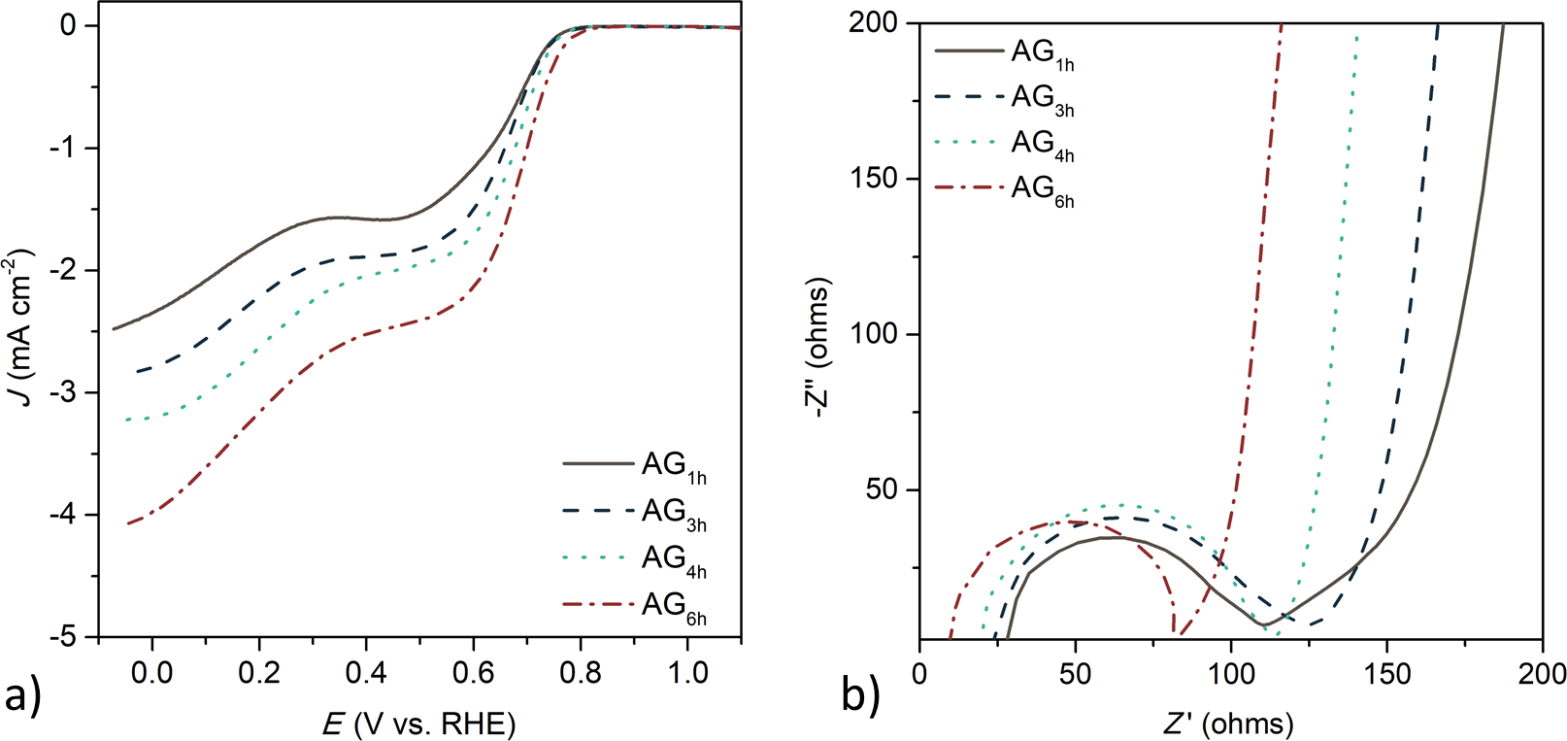
Linear sweep voltammetry recorded in an O_2_-saturated 0.1 mol L^−1^ KOH electrolyte at 1600 rpm (a) and Nyquist plot obtained from electrochemical impedance spectroscopy (b).

Linear sweep voltammograms (LSVs) recorded in an O_2_-saturated basic electrolyte at 1600 rpm and the Nyquist plot obtained from electrochemical impedance spectroscopy measurements are shown in Figure 2a and 2b, respectively.

**Figure 3:**
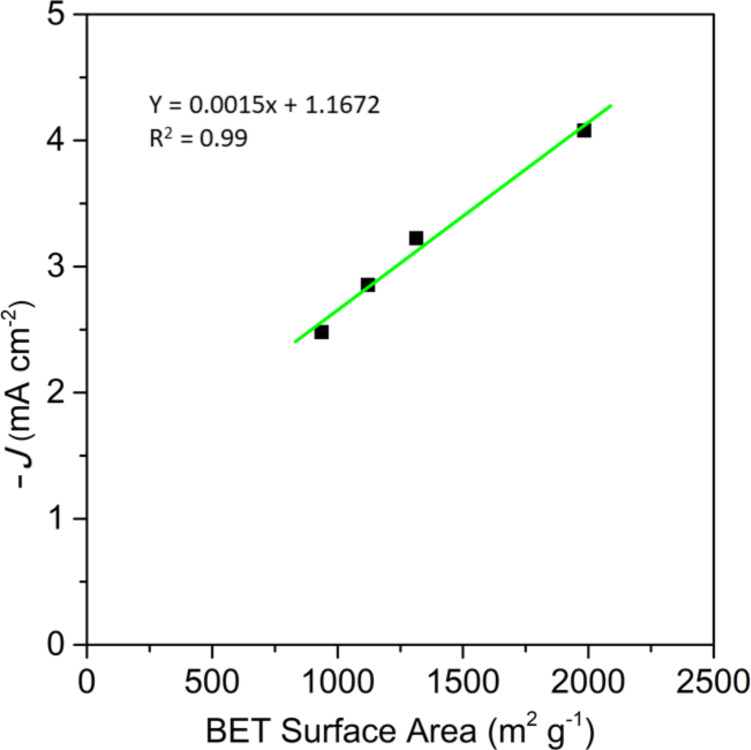
Relationship between BET surface area and the limiting current density of the activated samples.

To evaluate the performance of the prepared electrocatalysts, cyclic voltammetry (CV) and linear sweep voltammetry (LSV) were performed. LSV curves of the activated samples show two main differences: i) the onset potential shifts to more positive values by increasing the time of activation, which can be related to the more graphitic structure that is generated during activation; and ii) the value of the limiting current density increases with microporosity, which can be related to the more developed porous structure. These two effects can be confirmed by the electrochemical impedance spectroscopy measurements (Figure 2b). The Nyquist plot shows that a higher degree of activation results in a lower cell resistance and a smaller semicircle at high frequencies, indicating a lower charge transfer resistance, which allows the kinetics of the ORR to increase, and consequently, a higher onset potential is observed for sample AG_6h_ (the values of the onset potential are shown in Supporting Information File 1, Table S4). In addition, these results also suggest that a more developed microporous structure favors the electrolyte diffusion to the most electrochemically active pores, which also contributed to the ORR kinetics. Moreover, clear differences regarding ionic transportation are also observed at medium frequencies. Sample AG_1h_ shows a more defined Warburg impedance, indicating a higher resistance of the electrolyte ion diffusion into the porous structure, and hence, a lower value of limiting current density. These diffusion limitations are less evident for those samples with wider pore size, as pores act as diffusion channels favoring the kinetics of the ORR. In fact, a direct relationship was observed for microporosity and limiting current density for these carbon materials (Figure 3).

However, regardless of the time used for the activation, all samples show a LSV curve with a similar shape. The reduction reaction occurs at two different potentials (0.75–0.78 V and 0.30–0.33 V), indicating that the ORR mechanism proceeds via the two-electron pathway. These results are corroborated by the experiments performed with a rotating ring disk electrode, from which the production of hydrogen peroxide was evaluated. More than 18% of hydrogen peroxide was produced with AG_6h_ (Supporting Information File 1, Figure S2), which is not desirable for the ORR. These results indicate that further modifications of this sample are needed to enhance the performance of the biomass-derived carbons towards ORR.

### Effect of the surface chemistry

In order to improve the performance of glucose-derived activated carbons, the surface chemistry of the sample AG_6h_ (from now on simply named AG) was modified by applying different doping methods: ball milling and conventional mixing. In addition, such methods were also applied to a carbonized sample to evaluate the effect of the doping method according to the microporosity generated from the initial thermal treatment. Both activated and carbonized samples also underwent the ball milling method without nitrogen precursor to discriminate among the modifications resulting from ball milling and those due to the conjugation of ball milling and the nitrogen precursor. The results obtained from the elemental analysis are presented in Table 1.

**Table 1:** Chemical composition determined by elemental analysis.

Sample	Carbon (wt %)	Nitrogen (wt %)	Oxygen (wt %)	Hydrogen (wt %)

AG	97.3	–	2.4	0.3
AG_BM_	89.4	–	9.5	1.1
N-AG_BM_	87.3	4.3	7.0	1.4
N-AG_C_	90.1	4.1	4.9	0.9

CG	93.6	–	4.7	1.6
CG_M_	85.5	–	12.3	2.2
N-CG_BM_	82.9	6.9	8.8	1.4
N-CG_C_	83.1	6.2	9.2	1.5

As expected, all samples are mainly composed of carbon, where the percentage is slightly higher for activated samples than for carbonized materials, due to the higher temperature used for activation (900 °C). The application of ball milling in both activated and carbonized samples results in a noticeable increase in the oxygen content. This phenomenon may be due to the defects created in the carbon structure during the milling process that react with air and incorporate oxygen. This effect is also observed for N-doped samples by ball milling. However, doped samples undergo a second thermal treatment that partially removes the oxygen incorporated during the ball milling process, resulting in lower oxygen content. Nevertheless, although the amount of oxygen in samples doped by ball milling is lower than in undoped samples, the percentage detected is still significant. As for conventionally doped samples, the oxygen content is twice the oxygen found in the original samples. Regardless of the degree of activation of the samples, the amount of nitrogen incorporated by the ball milling method is similar to that obtained by the conventional method. However, the incorporation of oxygen and nitrogen in the activated structures is lower than in carbonized samples. Activation at high temperature results in a structure with higher chemical stability and lower amount of defects in which heteroatoms can be incorporated, and so materials with a lower degree of functionalization are obtained.

Further understanding of the functionalities of the carbon materials was achieved by analyzing their surface composition by X-ray photoelectron spectroscopy (XPS). The XPS spectra in the C 1s, O 1s and N 1s regions were deconvoluted to identify the types of functionalities present in the surface of the carbon materials. The deconvolution of the C 1s spectra for undoped samples presents five main peaks (Supporting Information File 1, Figure S3), representing the following by increasing binding energy: i) carbon sp^2^ (C=C, peak I) at 284.6 ± 0.1 eV; ii) carbon in phenol, alcohol, ether bonds (C–O, peak II) at 285.8 ± 0.2 eV; iii) carbonyl or quinone groups (C=O, peak III) at 287.2 ± 0.2 eV; iv) carboxyl groups (COOH, peak IV) at 288.9 eV ± 0.3; and v) the shake-up satellite due to π–π* transitions in aromatic rings (peak V) at 290.6 ± 0.5 eV [43]. Carbon sp^2^ (peak I) is an asymmetric peak consisting of a tail towards higher binding energies that represents ≈80% of surface carbon, which does not show significant differences for activated and carbonized samples. However, noticeable differences in the peaks attributed to carbon–oxygen bonds are observed due to the ball milling process and the doping methods applied. The peak attributed to carboxylic acids (peak IV) is significantly more pronounced for ball-milled samples (AG_BM_ and CG_BM_), suggesting that the increase of oxygen detected by elemental analysis (Table 1) was due to the formation of a large number of carboxylic acids, which further reinforces the possible reaction between the defects generated on the sample during the ball milling process and air moisture. The XPS spectra for the C 1s region of the doped samples exhibit the same five peaks as those observed for the undoped samples. However, peak II and peak III also have contributions of C–N and C=N interactions, respectively [15]. These phenomena result in a significantly higher contribution of these two peaks than those observed for the undoped samples. Additionally, peak IV in conventionally doped materials (N-AG_C_ and N-CG_C_) is more pronounced than that of their doped ball-milled counterparts. This peak can be assigned to sp^2^-hybridized carbons in a triazine aromatic ring (N–C=N) [30], which may result from the polymerization of melamine during the subsequent thermal treatment. This effect is less obvious in the spectra of the activated sample, as its higher chemical stability results in a lower degree of functionalization and, therefore, a lower formation of the triazine aromatic ring.

**Figure 4:**
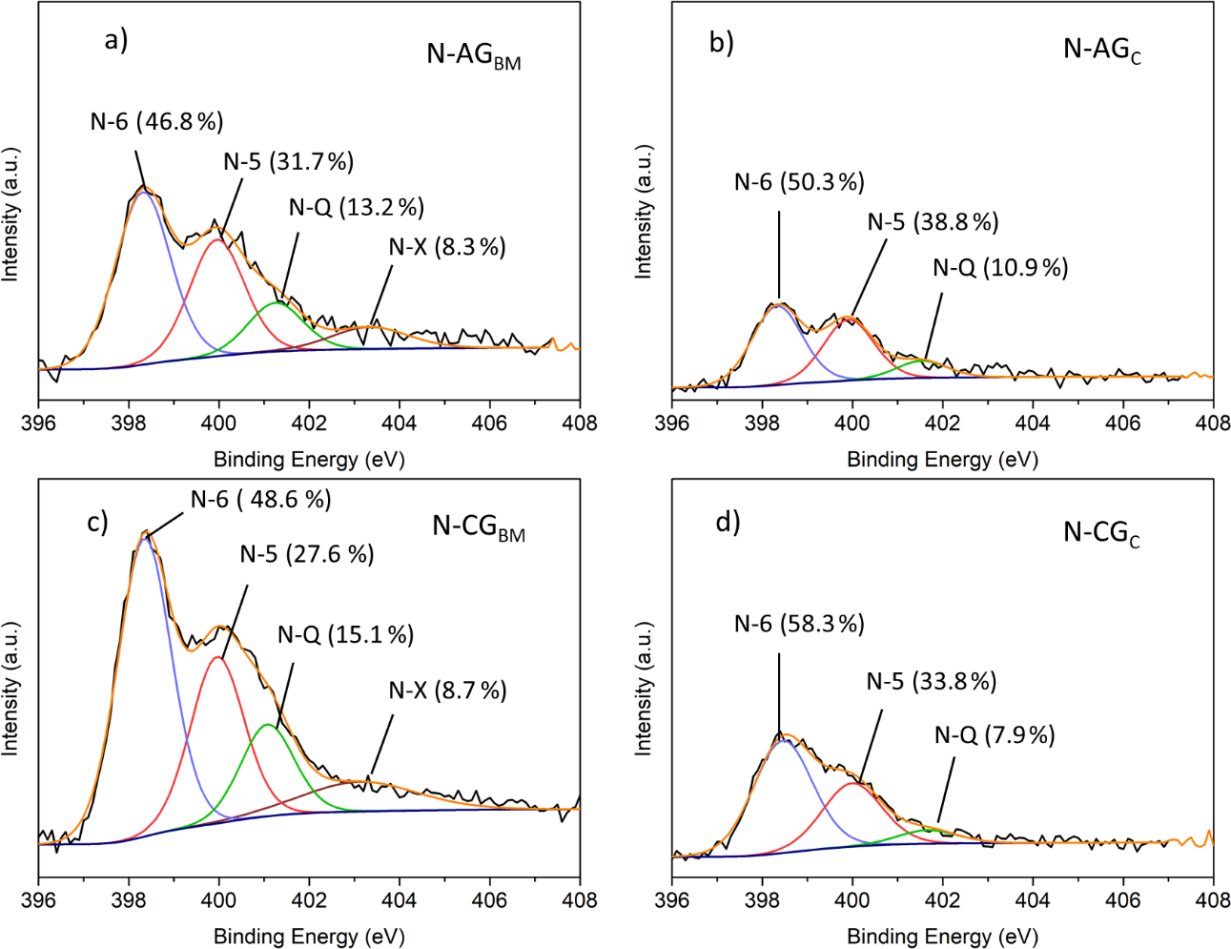
Deconvolution of the XPS N 1s spectra for N-AG_BM_ (a) N-AG_C_ (b), N-CG_BM_ (c) and N-CG_C_ (d).

The high-resolution N 1s spectra of doped samples was deconvoluted into three different peaks (Figure 4) representing the three major nitrogen groups, which are, by increasing binding energy: pyridinic at 398.4 ± 0.1 eV (N-6), pyrrolic at 400.0 ± 0.1 eV (N-5) and quaternary nitrogen at 401.4 ± 0.3 eV (N-Q) [12,26]. Additionally, another peak was observed at 403.2 ± 0.1 eV in samples N-AG_BM_ and N-CG_BM_, attributed to oxidized nitrogen groups (N-X) [26], suggesting that the ball milling process is related to the appearance of oxidized groups, as this peak is not observed for conventionally doped samples (sample N-AG_C_ and N-CG_C_). The ball milling method seems to modify the carbon structure promoting the contact between oxygen and nitrogen-containing species, which will react during the subsequent thermal treatment, oxidizing the nitrogen groups. In addition, differences in the contribution of each nitrogen group due to the doping method were also observed.

Samples doped via ball milling (Figure 4a and 4c) exhibit slightly lower percentages of pyridinic and pyrrolic groups than their conventionally doped counterparts (Figure 4b and 4d). Regarding quaternary nitrogen, significant differences were observed for both the doping method and the structure of the carbon. Activated samples exhibit similar contributions (13% and 11% for samples N-AG_BM_ and N-AG_C_, respectively), although ball milling seems to favor the incorporation of a slightly larger amount of N-Q. This effect is much more pronounced in carbonized samples that present larger differences as a function of the doping method: N-CG_BM_ incorporated almost twice N-Q groups as N-CG_C_. These results suggest that the ball milling process is more prone to incorporate quaternary groups as it creates defects in the activated and carbonized glucose structure, creating more sites to form quaternary structures inside the carbon matrix.

**Figure 5:**
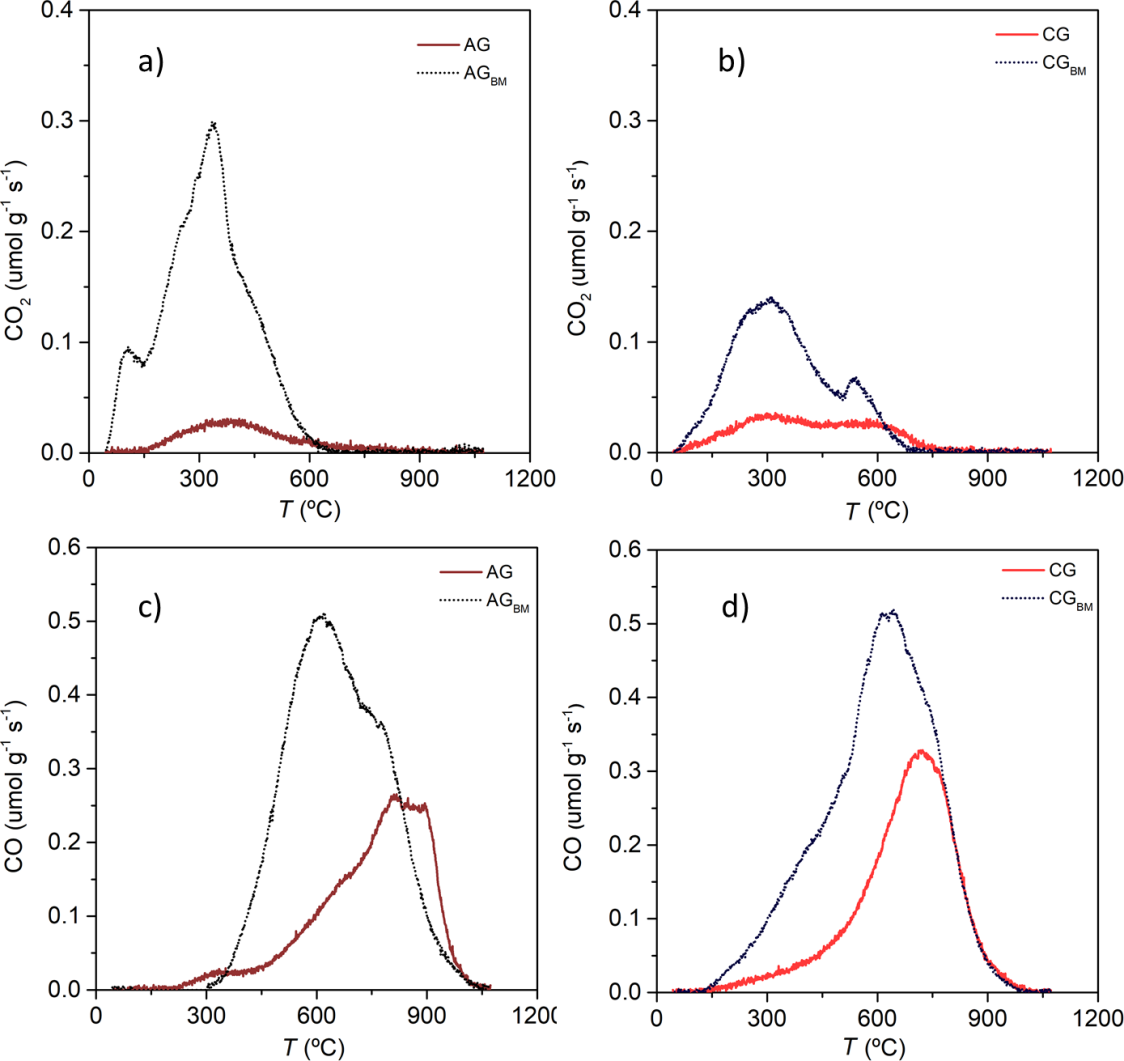
TPD profiles of CO_2_ for activated samples (a) and carbonized samples (b) and CO profiles of activated samples (c) and carbonized samples (d).

The XPS spectra in the O 1s region are shown in Supporting Information File 1, Figure S4. Three main oxygen peaks were identified, attributed to C=O bonds at 530.7 ± 0.3 eV (peak I), C–O groups at 532.0 ± 0.2 eV (peak II) and carboxylic acids at 533.3 ± 0.2 eV (peak III) [27]. Interesting differences are detected for N-doped carbons prepared by the ball milling method, as an additional peak is detected at higher binding energies corresponding to N–O–C bonds (peak IV), which is in agreement with the oxidized nitrogen peak also shown in the N 1s spectra. In addition, different contributions of the oxygen functionalities related to carbon bonds are presented, which is in agreement with those results obtained by the deconvolution of the C 1s spectra, especially for undoped ball-milled samples that exhibited a pronounced peak attributed to carboxylic acids. The O 1s spectra deconvolution does not clearly distinguish the contribution of the various surface groups due to overlaps in their binding energies. Therefore, the contribution of oxygen-containing surface groups in undoped samples was further analysed by TPD. The CO and CO_2_ measurements obtained for undoped activated and carbonized samples are presented in Figure 5, while the deconvolution of the profiles is shown in Figures S5, S6, S7 and S8 in Supporting Information File 1.

The amount of CO_2_ and CO released from ball-milled samples is significantly higher than that of their original counterparts. Analyzing the CO_2_ profiles of activated samples three peaks can be observed (Supporting Information File 1, Figures S5a and S6a), which could be attributed to carboxylic acids, anhydride and lactone groups, respectively [26,44]. The peaks presented for the ball-milled activated sample are much more intense, especially the carboxylic acid peak, which is in agreement with the results obtained from XPS. The deconvolution of the CO profiles (Supporting Information File 1, Figures S5b, S6b, S7b and S8b) of all samples showed the presence of three main peaks corresponding to anhydrides, phenols and carbonyl/quinone groups [26,44]. Ball-milled samples present a much more significant contribution of phenols, corroborating the hypothesis that ball milling generates oxygen functional groups with weaker bonds due to reaction with air moisture. It should also be noted that the temperature at which oxygen groups are released as CO_2_ and CO is higher for sample AG than for sample CG, confirming that the activated sample is more chemically stable.

**Figure 6:**
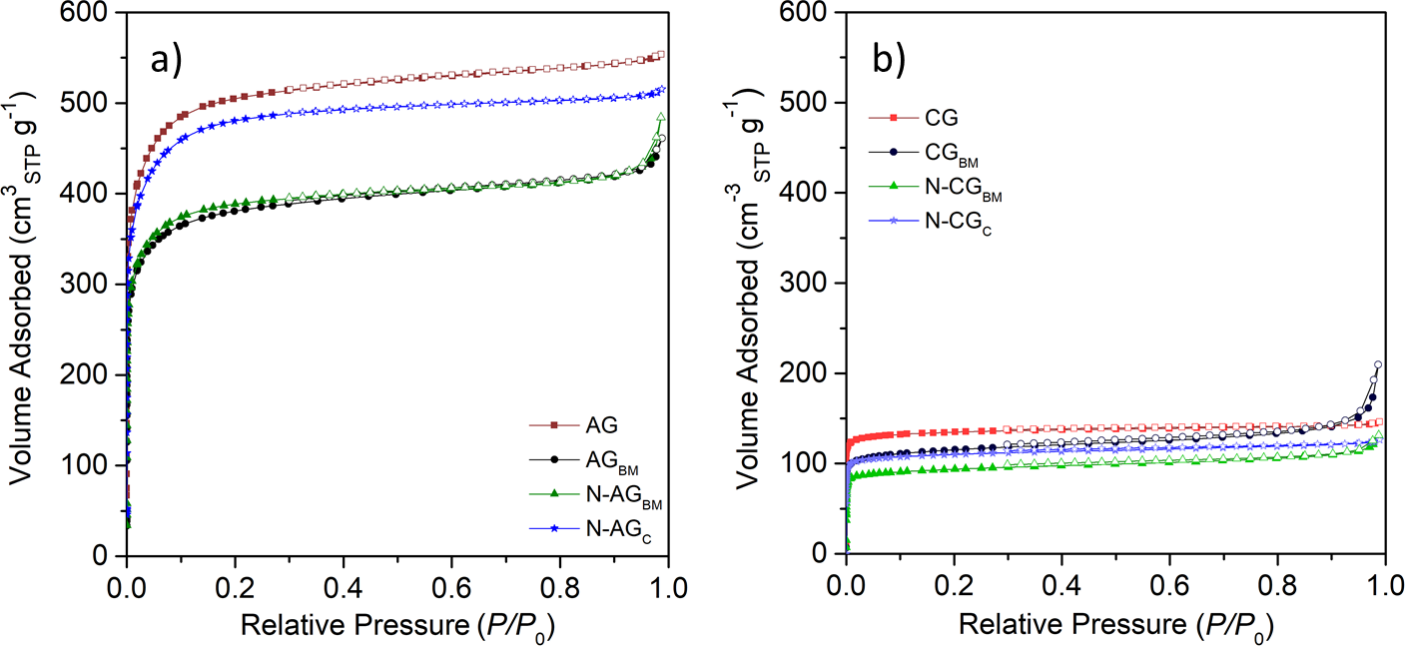
N_2_ adsorption/desorption isotherms at −196 °C for activated carbons (a) and carbonized carbons (b).

The doping methods employed may also have an effect on the textural properties, which, as explained above, may modify the electroactive character of the biomass-derived carbons. Therefore, to evaluate the modifications caused to the textural properties due to the surface chemistry changes, nitrogen adsorption–desorption isotherms were obtained (Figure 6). The pore size distributions determined by applying the QSDFT are shown in Supporting Information File 1, Figure S9.

Like activated samples shown in Figure 1, modified activated and carbonized carbon materials display type I isotherms, characteristic of microporous materials. The application of ball milling and the different doping methods to both activated and carbonized samples led to a decrease in the volume of nitrogen adsorbed at low relative pressure, resulting in materials with a smaller volume of micropores (Supporting Information File 1, Table S5) and lower surface area (a decrease of ≈500 and 100 m^2^ g^−1^ is observed after ball milling in the activated and carbonized samples, respectively). This decrease in microporosity suggests that the carbon material suffered changes in its structure due to the ball milling process, especially for the activated sample (Supporting Information File 1, Figure S10). This effect may be due to the loss of mass during the activation that results in materials with lower mechanical resistance than that of carbonized samples [45–46]. Moreover, ball-milled samples exhibit an increase of nitrogen adsorbed at high relative pressure (*P*/*P*_0_ > 0.9), suggesting the formation of macropores. The addition of nitrogen by conventional mixing the carbon materials with melamine also results in a decrease in the surface area. In this case, the decrease is analogous for both activated and carbonized sample, suggesting that the N-groups introduced into the structure are blocking some pores, preventing the access of N_2_ to the innermost pores of the carbon material during the isotherm measurements. The incorporation of nitrogen functionalities by using the ball milling process also modified the microporosity of the samples. However, differences are observed for the activated and carbonized sample. In the case of the activated sample, the incorporation of nitrogen does not seem to produce a noticeable blockage of the pores as previously observed for sample N-AG_C_, resulting in a material with a BET surface area similar to that obtained for sample AG_BM_, with ball milling being the predominant effect. In the case of sample N-CG_BM_, the ball milling process and the incorporation of nitrogen seem to have a synergistic effect, resulting in a decrease of the S_BET_ almost equivalent to the sum of that observed for sample CG_BM_ and N-CG_C_.

### Electrochemical measurements

**Figure 7:**
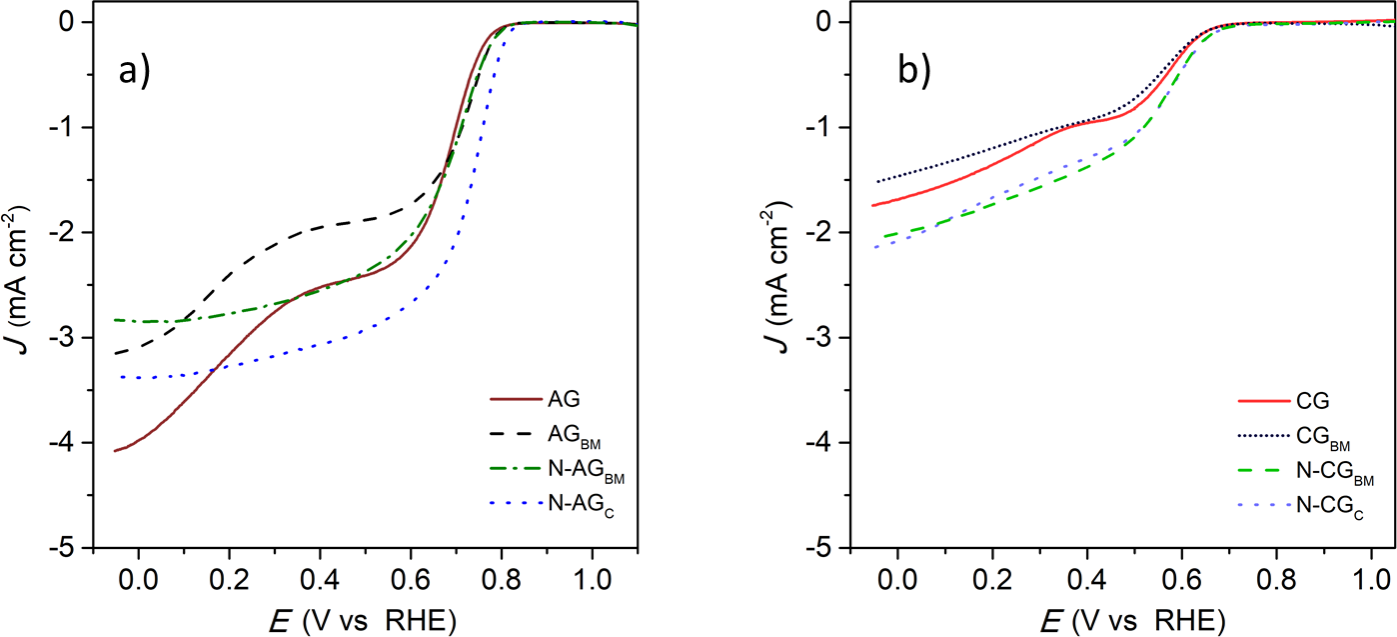
Linear sweep voltammetry recorded in an O_2_-saturated 0.1 mol L^−1^ KOH electrolyte at 1600 rpm for activated (a) and carbonized (b) samples.

Cyclic voltammograms recorded for activated samples and carbonized samples in a N_2_- and O_2_-saturated basic electrolyte at 5 mV s^−1^ are shown in Supporting Information File 1, Figure S11, while the LSV of activated and carbonized samples recorded in an O_2_-saturated basic electrolyte at 1600 rpm are shown in Figure 7a and 7b, respectively.

Cyclic voltammograms measured in O_2_-saturated electrolyte (Supporting Information File 1, Figure S11a) and LSV of activated samples show a reduction reaction peak starting at 0.78–0.82 V. This peak does not appear for N_2_-saturated cyclic voltammograms (Supporting Information File 1, Figure S11c), thus confirming that the catalytic activity of the prepared electrocatalysts exists at the mentioned potentials. Table 2 summarizes the electrochemical results of the samples.

**Table 2:** Electrochemical results of the synthesized samples.

Sample	Onset potential (V vs RHE)	Limiting current density (mA cm^−2^)	Electrons exchanged at 0.4 V vs RHE	H_2_O_2_ production (%)

AG	0.78	4.08	2.3	18
AG_BM_	0.79	3.15	2.0	21
N-AG_BM_	0.79	3.37	2.9	9
N-AG_C_	0.82	2.83	3.2	7

CG	0.64	1.74	1.8	–
CG_BM_	0.64	1.53	2.3	–
N-CG_BM_	0.67	2.15	2.1	–
N-CG_C_	0.67	2.05	2.1	–

The onset potential of sample AG slightly shifts to more positive values from 0.78 V to 0.79 V by ball milling the sample (AG_BM_) as shown in Figure 7a and Table 2. This increase may be due to the smaller particle size of the carbon powder obtained after ball milling, which could result in a material with higher electrical conductance, or due to the higher amount of oxygen [13]. The LSV of carbonized samples reveals that although sample CG_BM_ presents a much higher oxygen content, CG and CG_BM_ samples display the same onset potential (0.64 V), which suggests that the more positive onset potential of sample AG_BM_ is due to its higher conductance. Moreover, samples AG and AG_BM_ exhibit a second shoulder at more negative potentials, indicating that the ORR mechanism proceeds via the two-electron pathway producing hydrogen peroxide. This second reduction shoulder does not appear for the doped activated samples, suggesting that the mechanism of the reaction has shifted to the four-electron pathway. However, the incorporation of nitrogen atoms by ball milling the activated sample (N-AG_BM_) does not modify the onset potential when compared to the undoped counterpart (AG_BM_). This indicates that the simple incorporation of nitrogen atoms is not enough to increase the onset potential to more positive values for this type of carbon material. Contrary to activated samples, the incorporation of nitrogen atoms using the ball milling method in the carbonized sample (N-CG_BM_) slightly increases the onset potential regarding the undoped sample (CG_BM_) from 0.64 V to 0.67 V. Like in activated samples, this is probably due to the smaller particle size obtained after ball milling, which could result in a material with higher electrical conductance.

The onset potential of sample N-AG_C_ is shifted to more positive values in relation to AG (from 0.78 V to 0.82 V). AG and N-AG_C_ do not present significant differences in the nature and amount of oxygen groups at their surface (Supporting Information File 1, Figure S4), which suggests that the increase in the onset potential is due to the incorporation of nitrogen. However, sample N-AG_BM_ and N-AG_C_ present similar nitrogen content (Table 1), but different values of onset potential are obtained, suggesting that the percentage of incorporated nitrogen is not the key factor, but the type of nitrogen functionality. Likewise, the addition of nitrogen using the conventional method (N-CG_C_) also results in the same slight shift of the onset potential to more positive values in relation to CG and CG_BM_.

In order to further understand the effect of nitrogen functionalities in the ORR, doped samples must be thoroughly compared. A difference in the content of pyridinic nitrogen is observed for doped activated samples (39% vs 32% for N-AG_C_ and N-AG_BM_, respectively, Figure 4). Accordingly, the increase of the onset potential may be due to the presence of pyridinic nitrogen groups in the N-AG_C_ sample, which is in agreement with some studies in the literature [23]. The sample N-AG_C_ showed a higher value of the pyridinic-N/quaternary-N ratio than the sample N-AG_BM_ (3.6 vs 2.4, respectively) and a slightly higher value of the pyridinic-N/pyrrolic-N ratio (0.8 vs 0.7, respectively) which corroborates the theory that the increase of these two ratios is beneficial for the electrochemical activity of the prepared electrocatalysts [12]. This is not observed for carbonized samples as they do not present superior textural properties, compromising some of the effects that nitrogen could have on the carbon as a catalyst.

**Figure 8:**
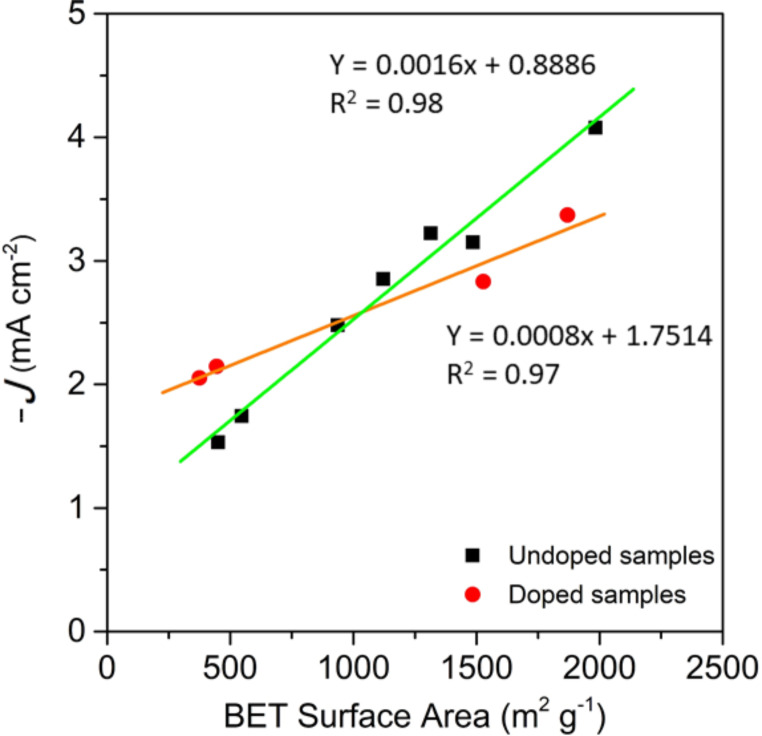
Relationship between BET surface area and limiting current density of undoped and doped samples.

Information about the limiting current density can also be obtained from the LSV curves. As previously observed for activated samples at different times, there is a direct relationship between microporosity and the limiting current density (Figure 3). This trend is also observed for sample group AG, AG_BM_, CG and CG_BM_ and for samples N-AG_C_, N-AG_BM_, CG_C_ and N-CG_BM_ (Figure 8).

These results indicate that the effect of microporosity cannot be compared between doped and undoped samples, suggesting that the limiting current density does not only depend on microporosity, but also on the surface chemistry of the samples.

Significant differences in the shape of the LSVs shown in Figure 7 are also observed due to the incorporation of nitrogen, which is related to the reaction mechanism. At lower potential, all activated samples present more than three electrons exchanged during the reduction reaction (Supporting Information File 1, Figure S12a). Regarding carbonized samples (Supporting Information File 1, Figure S12b), the number of electrons of CG and CG_BM_ represents a two-pathway mechanism at all potentials applied, unlike for the AG electrocatalysts. The electron exchange in the AG sample is reduced to two as the potential increases. The same effect was registered for the AG_BM_ sample that, even with a slightly inferior number of electrons exchanged at the lowest potential, the number of electrons also decreases to two as the potential applied increases. The incorporation of nitrogen atoms with the ball milling method (N-AG_BM_) mitigated this effect, as the number of electrons exchanged during the oxygen reduction reaction stays at approximately three electrons, demonstrating that nitrogen incorporation helps to stabilize the number of electrons for a larger potential range. The conventionally doped sample (N-AG_C_) shows the same pattern as the ball-milled doped sample, but its electron exchange stabilized slightly closer to the four-electron mechanism, probably due to the higher catalytic performance of pyridinic nitrogen groups, which maintains the reaction mechanism closer to a four-electron pathway. On the other hand, the decrease of the quaternary-N/pyrrolic-N ratio (from 0.28 to 0.22 for N-AG_BM_ and N-AG_C_, respectively) resulted in an increase of the number of electrons involved, which is in agreement with recent studies [12]. Unlike in activated samples, the addition of nitrogen atoms through ball milling (N-CG_BM_) does not result in an increase of electrons exchanged or in the stabilization of that number throughout the different applied potentials. This sample has a high quaternary-N/pyrrolic-N ratio (0.31), which does not favor the four-electron pathway.

Carbonized samples present a mechanism close to two-electrons throughout all potentials. Without a considerable number of electrons exchanged during the ORR, the nitrogen atoms do not seem to improve their stability to stay closer to a four-electron pathway. However, the conventionally doped sample (N-CG_C_) displays a slight increase of electron exchange at low potentials, but still shifts to a two-electron pathway with increasing applied potential. The slight increase of the electrons may be related to the very low quaternary-N/pyrrolic-N ratio (0.13), which is reported to influence the number of electrons. The fact that the addition of nitrogen functionalities on carbonized samples does not seem to enhance the electroactivity as for activated samples indicates that it is fundamental to design both the surface chemistry and the textural proprieties of the carbon materials, as the modification of a single parameter is not enough to obtain acceptable electrocatalysts for ORR.

The approximation to a four-electron pathway with the modification of the surface chemistry is important to reduce the amount of hydrogen peroxide produced. The production of H_2_O_2_ was only determined for activated samples, since they have a mechanism closer to four-electron, whereas carbonized samples have a two-electron mechanism that would result in high amounts of this intermediate product. The improvement of the reaction mechanism of the modified activated carbon materials led to a decrease in the production of H_2_O_2_ from 18% with sample AG to 7% with N-AG_C_ (Supporting Information File 1, Figure S13). These results show that the proper combination of high microporosity associated with high pyridinic-N/quaternary-N ratio and low quaternary-N/pyrrolic-N ratio is essential to enhance the electrochemical performance of the developed electrocatalysts.

## Conclusion

In this study, the effect of microporosity of glucose-derived carbon materials on the catalytic activity towards ORR was demonstrated. The increase of microporosity led to an increase of the limiting current density and a slight increase of the onset potential, thus playing a key role in the ORR. The incorporation of nitrogen functionalities by employing different doping methods was also investigated. The amount of nitrogen incorporated was similar for all methods. The ball milling doping method led to a higher content of quaternary nitrogen and to the formation of oxidized nitrogen, while conventional doping favored the incorporation of pyridinic and pyrrolic functionalities. The results obtained reveal that the content of nitrogen is not as important as the type of functional groups incorporated for improving the performance of carbon materials towards ORR. In fact, a relationship between the nitrogen functionalities and the electroactivity of the biomass-derived carbons has been determined. It has been observed that a higher pyridinic-N/quaternary-N ratio favors the onset potential, while a lower quaternary-N/pyrrolic-N ratio favors the number of electrons exchanged during ORR. However, these results are only significant for highly microporous materials, demonstrating that the adequate combination of textural and chemical properties is essential for improving the electroactivity of biomass-derived carbons. In fact, the combination between high surface area, high pyridinic-N/quaternary-N ratio and low quaternary-N/pyrrolic-N ratio resulted in a material with an onset potential value of 0.82 V, a stable number of electrons involved in the reaction mechanism close to four throughout the studied potential range and a production of H_2_O_2_ lower than 7%.

## Experimental

### Preparation of carbon materials

Carbon materials were prepared from an initial solution of glucose (HiMedia, >99%) and deionized water (produced by filtration through inverse osmose by a Panice device) in a 1:6 ratio. The solutions were mixed and then closed in a teflon-lined stainless steel autoclave and hydrothermally carbonized during 12 h at 180 °C. The obtained material was washed with deionized water and dried at 100 °C overnight. The dried material was then activated under a CO_2_ flow of 80 cm^3^ min^−1^ g^−1^ at 900 °C for 1, 3, 4 and 6 h. The samples were labelled AG_X_, where X is the hours used for activation.

The sample activated for 6 h was also doped with nitrogen by using melamine (≥99%, Sigma-Aldrich) as a precursor. Two different approaches were studied: i) the activated carbon material was mixed with melamine by ball milling and ii) the activated carbon material was manually mixed with melamine, henceforth referred to as the conventional method. The ball milling process was performed in an enclosed flask with two zirconia balls at 15 Hz frequency during 4 h using a Retsch MM200 device. Regardless of the doping method all samples underwent a subsequent thermal treatment under a N_2_ atmosphere for 2 h at 700 °C to force the decomposition of melamine and to incorporate nitrogen atoms into the carbon structure. Additionally, a carbonized sample was prepared under a N_2_ flow of 150 cm^3^ min^−1^ at 700 °C for 2 h, to isolate the effect of functionalization with respect to the surface area. This sample also underwent the same two doping methods as the activated sample. All treatments were carried out in a vertical furnace with a fixed heating rate of 10 °C min^−1^. The samples were labelled XG_Y_ and N-XG_Y_ for undoped and N-doped samples, respectively, where X can assume the form of A for activated samples and C for carbonized samples and Y is represented by BM for ball milled samples and C for conventionally doped samples. Activated and carbonized samples were also ball-milled in the absence of any precursor for comparison under the same conditions as in doped samples.

### Materials characterization

The textural characterization was carried out by N_2_ adsorption at −196 °C performed in a Quantachrome Autosorb iQ automated gas sorption analyzer. All samples were degassed under vacuum at 150 °C for 12 h before the analysis. The specific surface area (S_BET_) was determined according to the Brunauer–Emmett–Teller (BET) equation, the total pore volume (*V*_p_) was calculated as the volume of nitrogen adsorbed at the saturation point (relative pressure of 0.99) and the micropore volume (*V*_DR_) was evaluated by the Dubinin–Radushkevich method.

The chemical composition of the samples was determined by elemental analysis. Carbon, hydrogen and nitrogen (C, H and N) were determined in a Vario micro cube analyzer (Elementar GmbH), by combustion of the sample at 1050 °C. The oxygen content was determined using a rapid oxy cube analyzer (Elementar GmbH) in which the sample underwent pyrolysis at 1450 °C. Each sample was analysed in triplicate. X-ray photoelectron spectroscopy (XPS) was employed to study the surface chemical composition of the samples. The analyses were carried out in a Kratos AXIS Ultra HAS spectrometer using monochromatic Al Kα radiation (1486.7 eV) at 15 kV (90 W), in fixed analyzer transmission mode, performing a pass energy of 80 eV for the general spectra and 40 eV for regions of interest. Temperature programmed desorption (TPD) was performed to determine and quantify the surface oxygenated groups of the samples by using an Altamira Instruments AMI-300 device. The samples were heated with a 10 °C min^−1^ ramp until 1050 °C. At the end of each analysis, a calibration of the CO and CO_2_ content was carried out, allowing the quantification of the TPD profiles.

### Electrochemical characterization

The electrochemical measurements were performed on a PGSTAT 302N potentiostat/galvanostat by using a three-electrode cell configuration. Ag/AgCl (KCl 3 M) and a glassy carbon rod were used as reference and counter electrode, respectively. Working electrodes were prepared by depositing a suspension of the carbon samples on a glassy carbon rotating disk electrode (3 mm of diameter, Metrohm). These suspensions were prepared by dispersing 1 mg of the prepared samples in a solution containing 220 µL of ultrapure water (Millipore), 142 µL of ethanol (≥99%,Valente e Ribeiro) and 96 µL of nafion (5 wt %, Sigma-Aldrich). The suspension was sonicated for 30 min until a homogeneous dispersion was obtained. The mass loading of all samples was ≈0.1 mg cm^−2^. A rotation speed controller allowed the rotation of the working electrode to be adjusted according to the assessments being done.

The experiments were carried out at room temperature in a 0.1 mol L^−1^ KOH solution saturated with N_2_ or O_2_ for 30 min before the cyclic voltammetry (CV) and linear sweep voltammetry (LSV) were performed. The CVs measurements were accomplished at a scan rate of 5, 20, 60 and 100 mV s^−1^ and the LSVs at a scan rate of 5 mV s^−1^ with a rotation speed range from 400 to 3000 rpm, both within a 1.2 V to −0.1 V potential range (vs RHE). The measured current was determined by subtracting the current obtained from the electrolyte saturated with N_2_ from the current measured in the O_2_-saturated electrolyte. Electrochemical impedance spectroscopy (EIS) was also applied to the fully discharged cell at 0 V in the frequency region of 10 kHz to 10 mHz with an AC amplitude of 10 mV. EIS was performed in the same type of cell, with N_2_-saturated electrolyte (KOH 0.1 mol L^−1^) and with no rotation.

The current density at the disk can be expressed by the Koutecký–Levich equation:

[1] $$\frac{1}{j}=\frac{1}{j_{\text{L}}}+\frac{1}{j_{\text{k}}}=\frac{1}{B\omega^{1/2}}+\frac{1}{j_{\text{k}}},$$

where *j* is the measured current density (mA·cm^−2^), *j*_L_ is the O_2_ diffusion-limited current density (mA cm^-2^), *j*_k_ is the kinetic current density (mA cm^-2^), ω is the electrode rotation rate (rpm) and *B* represents the Levich constant related to the diffusion limiting current density given by Equation 2.

[2] $$B=0.2nFD^{2/3}\nu^{-1/6}C$$

In Equation 2, *F* is the Faraday constant (96 486 C mol^−1^), *D* is the diffusion coefficient of O_2_ (1.95 × 10^−5^ cm^2^ s^−1^), ν is the kinematic viscosity of the electrolyte (0.008977 cm^2^ s^−1^) and *C* is the bulk concentration of O_2_ (1.15 × 10^−3^ mol L^−1^).

The number of electrons *n* was calculated at different potentials for each LSV recorded by applying Equation 1 and Equation 2.

The percentage of hydrogen peroxide (H_2_O_2_) produced during the ORR was also measured by using a rotating ring disk electrode (5 mm diameter, 24.9% collection efficiency, Methrom) as a working electrode, which was prepared by depositing a solution of the carbon sample on the disk area. The dispersions were prepared as detailed above and the mass loading was fixed at ≈0.1 mg cm^−2^. The H_2_O_2_ percentage was calculated by Equation 3.

[3] $${\text{H}}_{\text{2}}{\text{O}}_{\text{2}}\text{(\%)}=200\times \frac{I_{\text{R}}/N}{I_{\text{D}}+I_{\text{R}}/N}$$

In Equation 3, *I*_R_ is the ring current density (mA cm^−2^), *I*_N_ is the disk current density of the disk (mA cm^−2^) and *N* is the collection efficiency (0.249).

## Supporting Information

File 1 Characterization of the carbon materials, electrochemical assessments and relations resulting from this work.
