# Supplementary material for: Glucose-derived carbon materials with tailored properties as electrocatalysts for the oxygen reduction reaction
Source: Beilstein J Nanotechnol. 2019 May 21;10:1089–102. doi: 10.3762/bjnano.10.109 (PMC6541360; doi:10.3762/bjnano.10.109)
Supplement: File 1 — Characterization of the carbon materials, electrochemical assessments and relations resulting from this work. [file Beilstein_J_Nanotechnol-10-1089-s001.pdf]

BEILSTEIN JOURNAL OF NANOTECHNOLOGY

Supporting Information

for

Glucose-derived carbon materials with tailored properties as electrocatalysts for the oxygen reduction reaction

Rafael Gomes Morais, Natalia Rey-Raap, José Luís Figueiredo
and Manuel Fernando Ribeiro Pereira

Beilstein J. Nanotechnol. **2019,** *10,* 1089–1102. doi:10.3762/bjnano.10.109

Characterization of the carbon materials, electrochemical assessments and relations resulting from this work

License and Terms: This is a supporting information file under the terms of the Creative Commons Attribution License (http://creativecommons.org/licenses/by/4.0). Please note that the reuse, redistribution and reproduction in particular requires that the authors and source are credited.
The license is subject to the *Beilstein Journal of Nanotechnology* terms and conditions: (https://www.beilstein-journals.org/bjnano)

Effect of microporosity

Table S1: Textural parameters of samples activated during different times.

**Sample**	**S_{BET} (m^2 g^{-1})**	**V_p (cm^3 g^{-1})**	**V_{DR} (cm^3 g^{-1})**
AG_{1h}	936	0.38	0.35
AG_{3h}	1121	0.44	0.43
AG_{4h}	1314	0.54	0.52
AG_{6h}	1984	0.85	0.82

Table S2: Elemental analysis of samples activated during different times.

**Sample**	**Carbon (wt %)**	**Oxygen (wt %)**	**Hydrogen (wt %)**
AG_{1h}	96.3	3.3	0.4
AG_{3h}	96.6	3.0	0.4
AG_{4h}	96.8	2.8	0.4
AG_{6h}	97.3	2.4	0.3

Table S3: CO and CO_2 amounts obtained from TPD measurements of AG_{1h} and AG_{6h}

**Sample**	**CO (μmol g^{-1})**	**CO_2 (μmol g^{-1})**	**CO/CO_2**	**Oxygen (%)**
AG_{1h}	1111	172	6.5	2.3
AG_{6h}	971	136	7.2	2.0

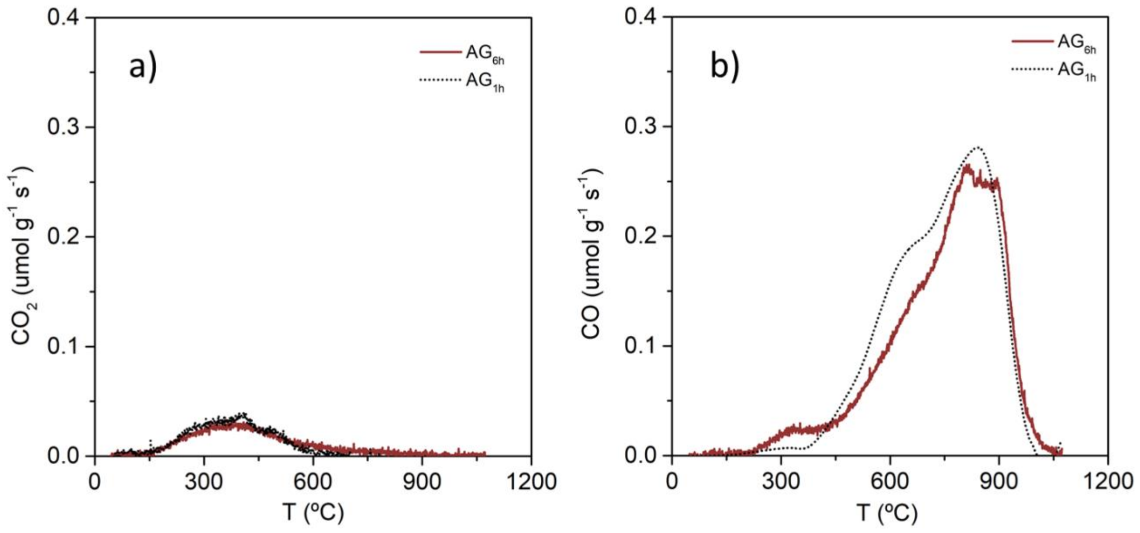

Figure S1: TPD profiles of CO_2 (a) and CO (b) for AG_{6h} and AG_{1h}.

Table S4: Onset potential of samples activated during different times.

Sample	Onset Potential (V)
AG_{1h}	0.75
AG_{3h}	0.75
AG_{4h}	0.76
AG_{6h}	0.78

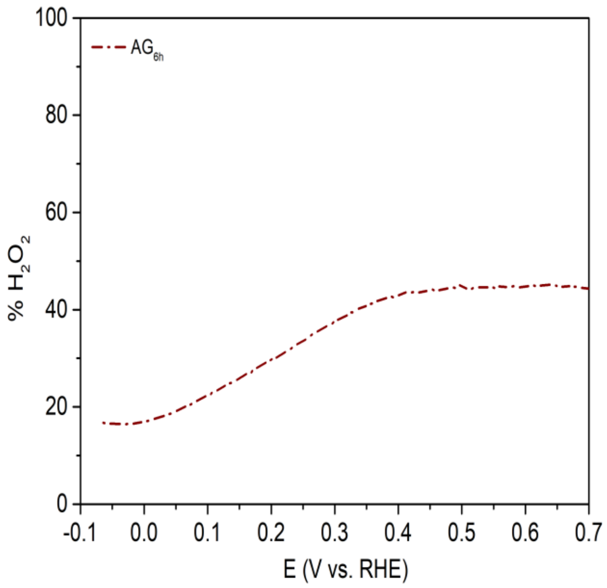

Figure S2: Hydrogen peroxide production for sample AG_{6h}.

Effect of the surface chemistry

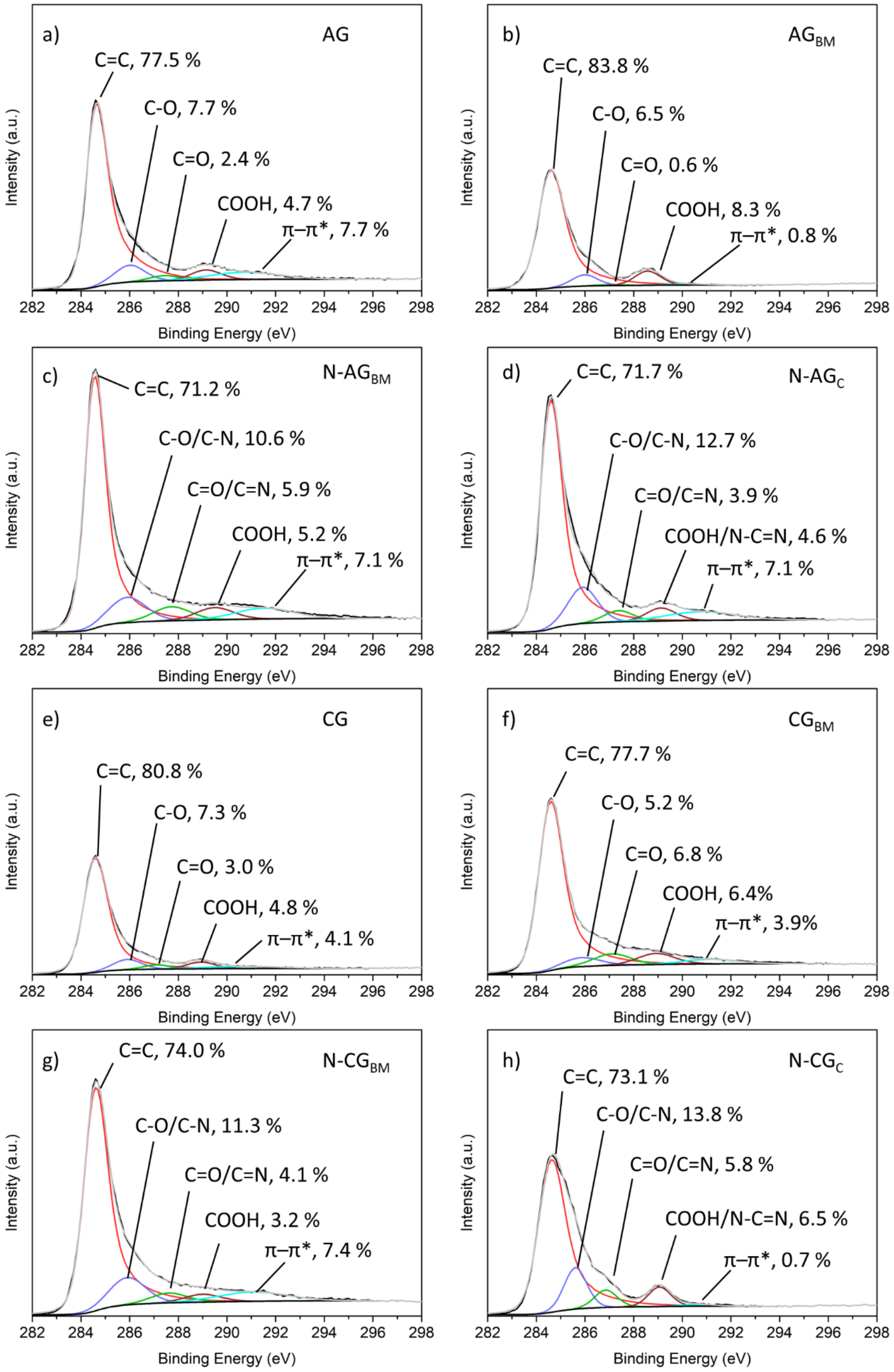

Figure S3: C 1s spectra deconvolution of AG (a), AG_{BM} (b), N-AG_{BM} (c), N-AG_{C} (d), CG (e), CG_{BM} (f), N-CG_{BM} (g), N-CG_{C} (h).

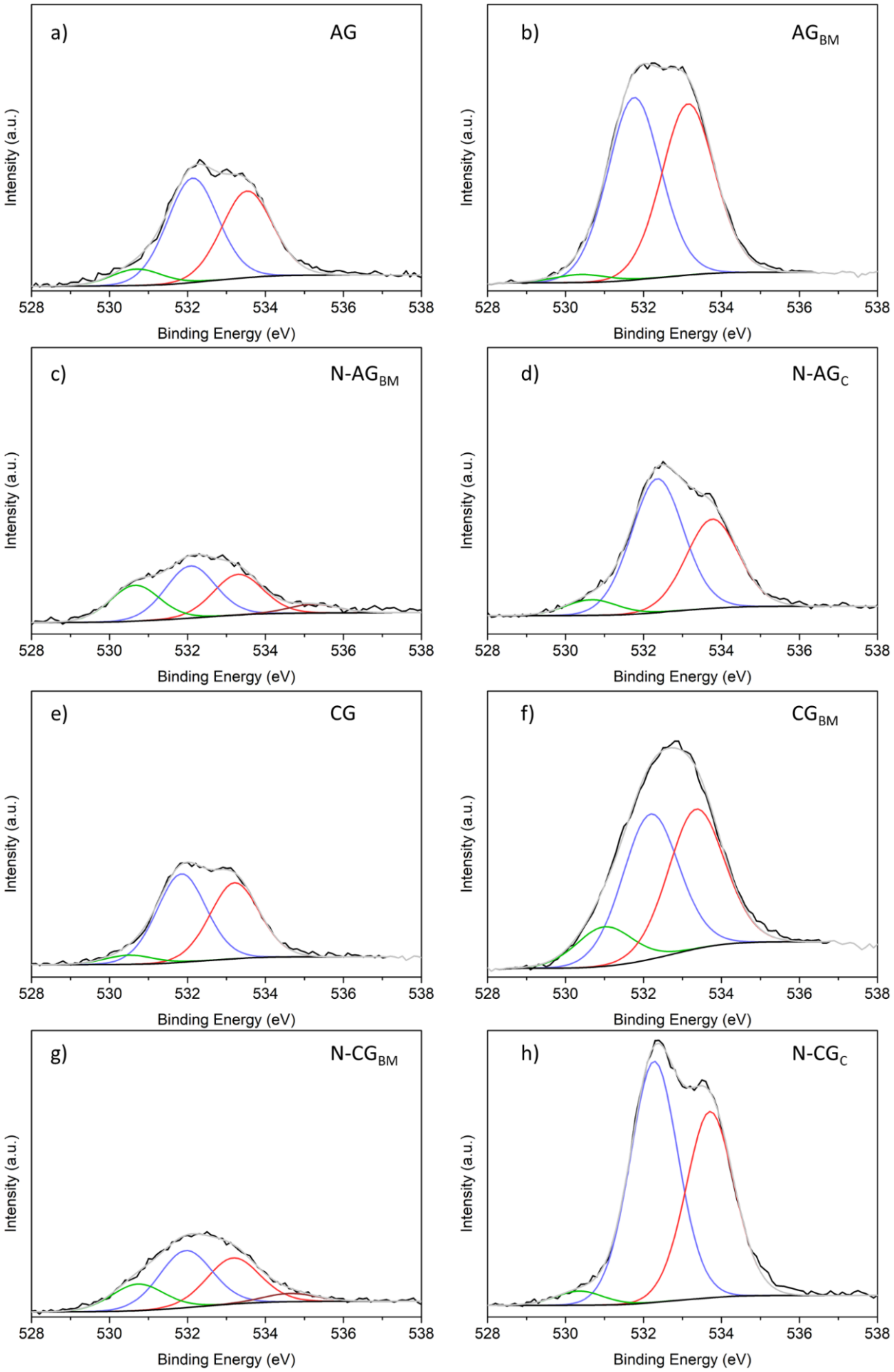

Figure S4: O 1s spectra deconvolution of AG (a), AG_{BM} (b), N-AG_{BM} (c), N-AG_{C} (d), CG (e), CG_{BM} (f), N-CG_{BM} (g), N-CG_{C} (h).

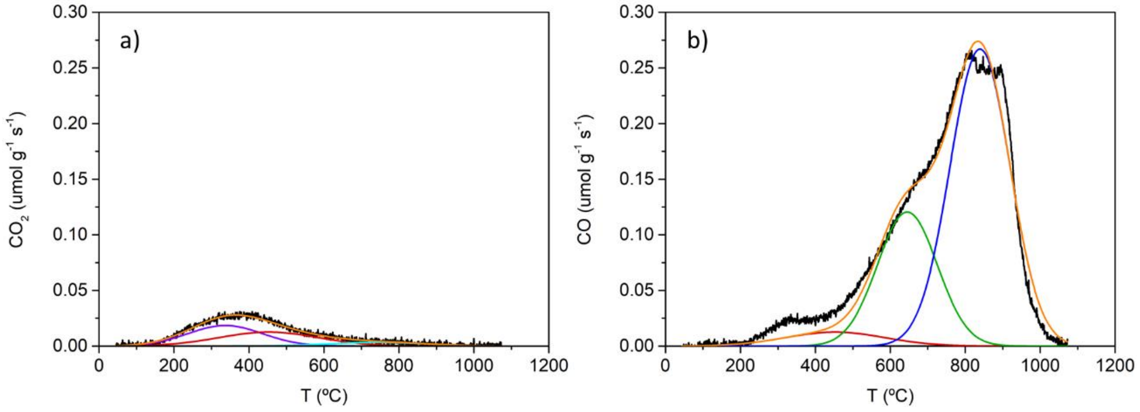

Figure S5: TPD profile deconvolution of AG for CO_2 (a) and CO (b).

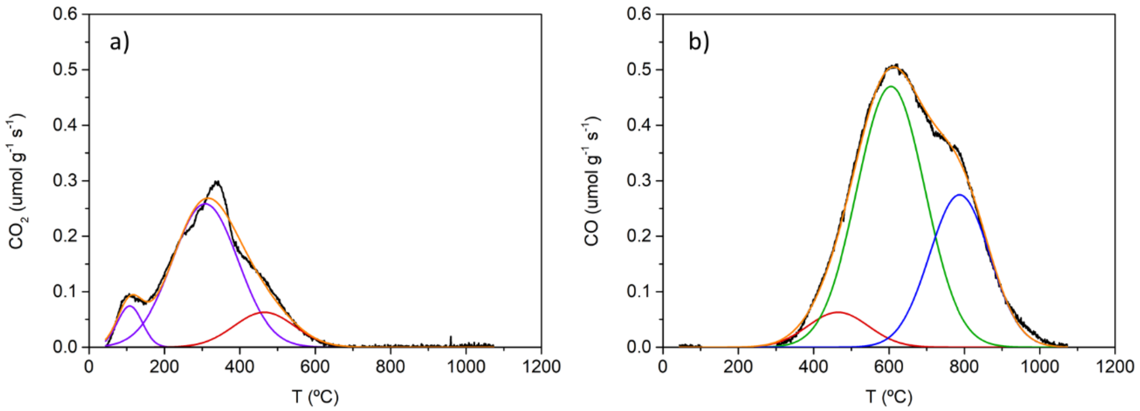

Figure S6: TPD profile deconvolution of AG_{BM} for CO_2 (a) and CO (b).

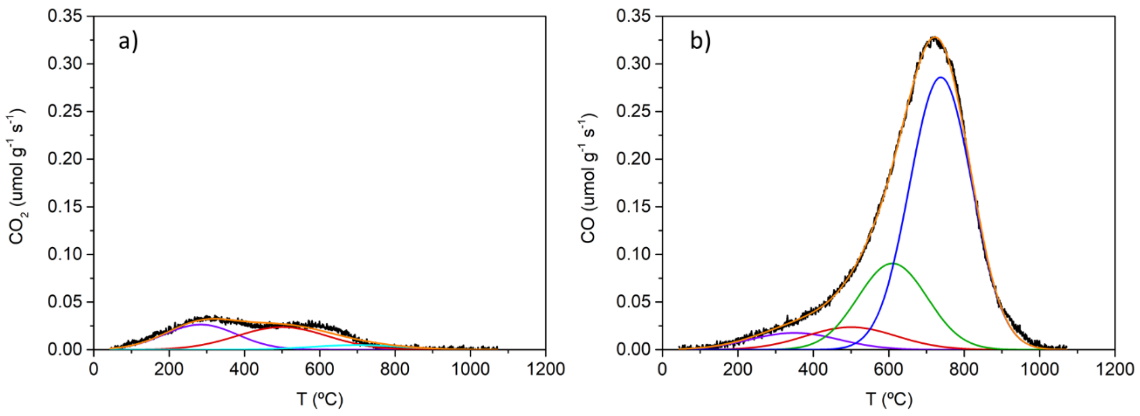

Figure S7: TPD profile deconvolution of CG for CO_2 (a) and CO (b).

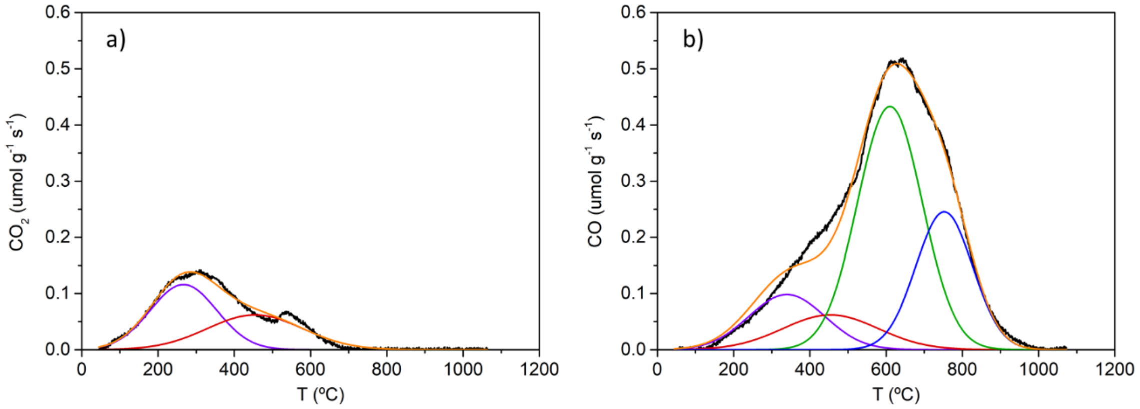

Figure S8: TPD profile deconvolution of CG_{BM} for CO_2 (a) and CO (b).

The activated sample (AG) presents three broad peaks in the CO_2 profile (Figure S5a), at 340 ºC, 450 ºC and 740 ºC, which could be attributed to carboxylic acids, anhydride and lactone groups, respectively. AG_{BM} also presents the carboxylic acids and anhydride peaks (Figure S6a). However, its CO_2 profile closes at lower temperatures than the CO_2 profile of AG, thus almost not presenting the peak attributed of lactones. The CO_2 profile of sample CG (Figure S7a) also presents the same three peaks as the activated sample. Similar to activated samples, the modification of the structure by the ball milling method (Figure S8a) results in an almost depletion of the lactone group and in a significant increase of the carboxylic acids, once again corroborating the XPS results. Since lactones are cyclic esters of carboxylic acids, the defects created by ball milling may be opening the cycle creating the opportunity for the chain to react with air moisture and thus originating carboxylic functional groups. As a result of the decrease of lactones detected from CO_2 release, an increase of carboxylic acids occurs.

The CO profile of sample AG (Figure S5b) presents three main peaks corresponding to anhydrides, phenols and carbonyl/quinone groups at temperature ranges of 450-470 ºC, 600-650 ºC and 790-840 ºC, respectively. These three groups are also present in sample AG_{BM} (Figure S6b) but with a much more significant contribution of phenols, corroborating the hypothesis of ball milling generating oxygen functional groups with weaker bonds due to reactions with air moisture. This

same effect is observed for carbonized samples (Figure S7b and Figure S8b for CG and CG_{BM}, respectively). Nevertheless, it should be noted that the temperature at which oxygen groups are released as CO_2 and CO is higher for sample AG than for sample CG, confirming that the activated sample is more chemically stable.

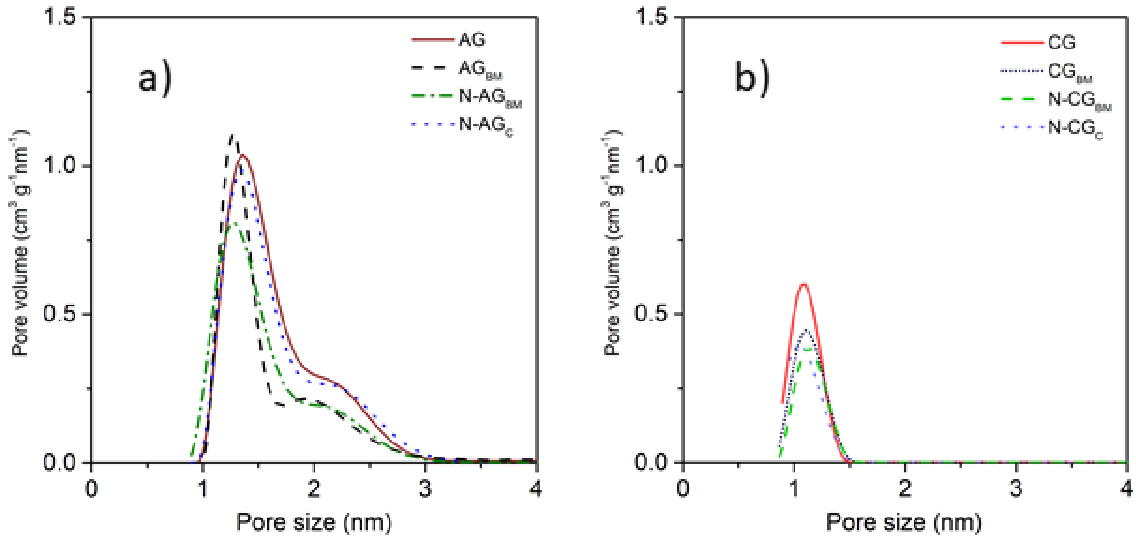

Figure S9: Pore size distributions for activated samples (a) and carbonized samples (b).

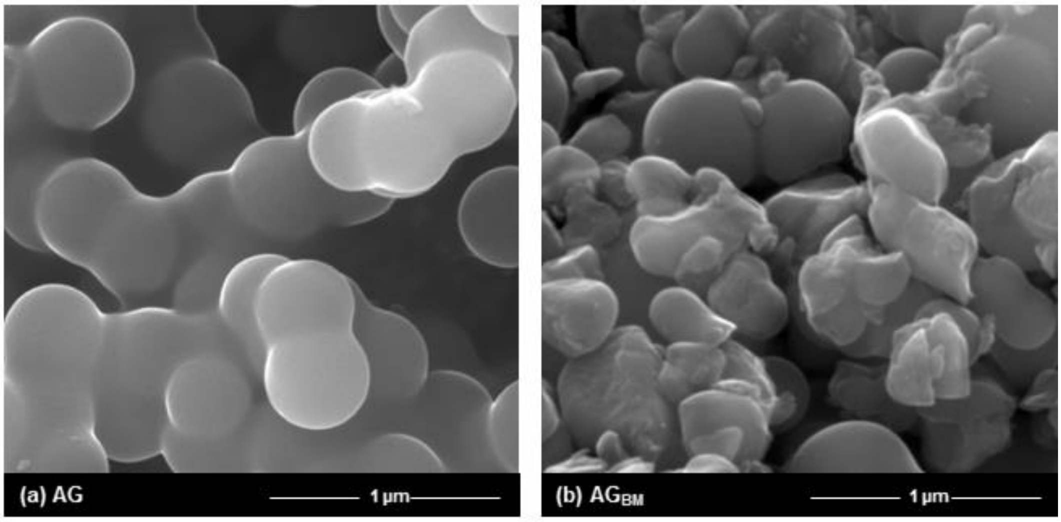

Figure S10: SEM images of activated glucose before (a) and after (b) ball-milling.

Table S5: Textural characterization of activated and carbonized samples.

Sample	S_{BET} (m^2 g^{-1})	V_p (cm^3 g^{-1})	V_{DR} (cm^3 g^{-1})
AG	1984	0.85	0.82
AG_{BM}	1486	0.71	0.54
$N\text{-}AG_C$	1870	0.79	0.68
$N\text{-}AG_{BM}$	1528	0.75	0.55
CG	547	0.23	0.20
CG_{BM}	451	0.32	0.17
$N\text{-}CG_C$	445	0.20	0.17
$N\text{-}CG_{BM}$	375	0.20	0.15

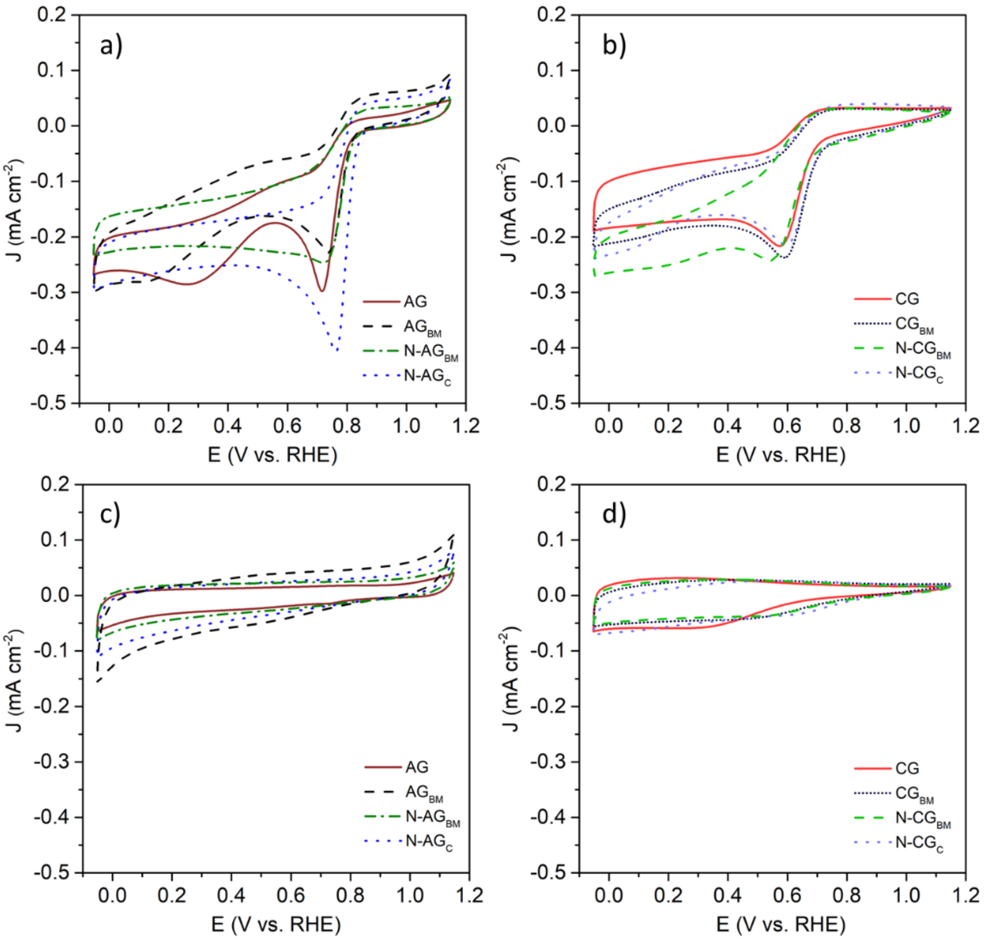

Figure S11: O_2-saturated cyclic voltammograms for activated samples (a) and carbonized samples (b) and N_2-saturated cyclic voltammograms for activated samples (c) and carbonized samples (d).

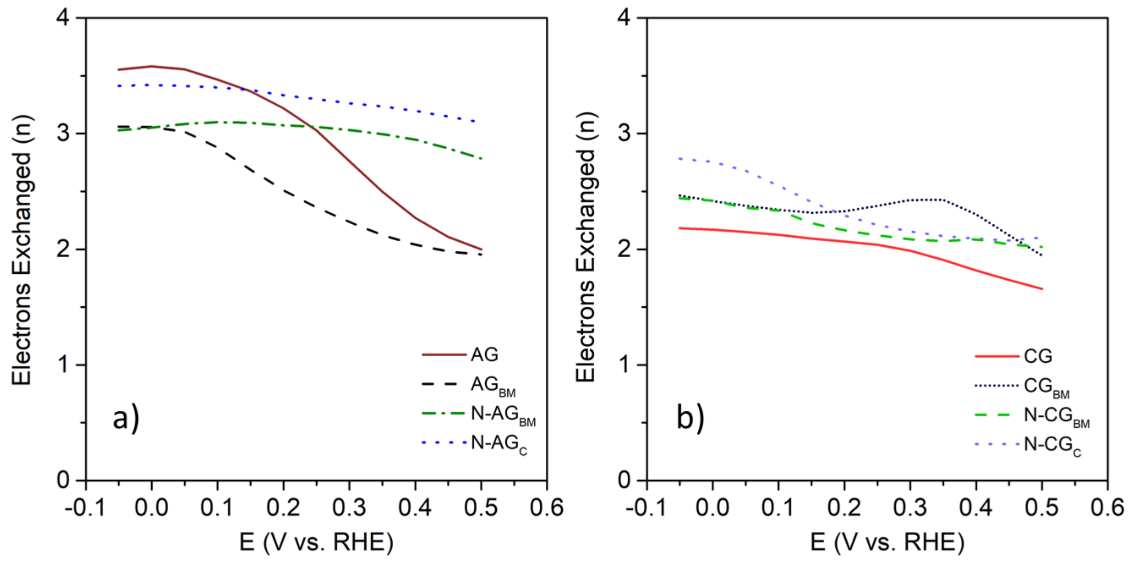

Figure S12: Number of electrons exchanged for activated samples (a) and carbonized samples (b) at different potentials.

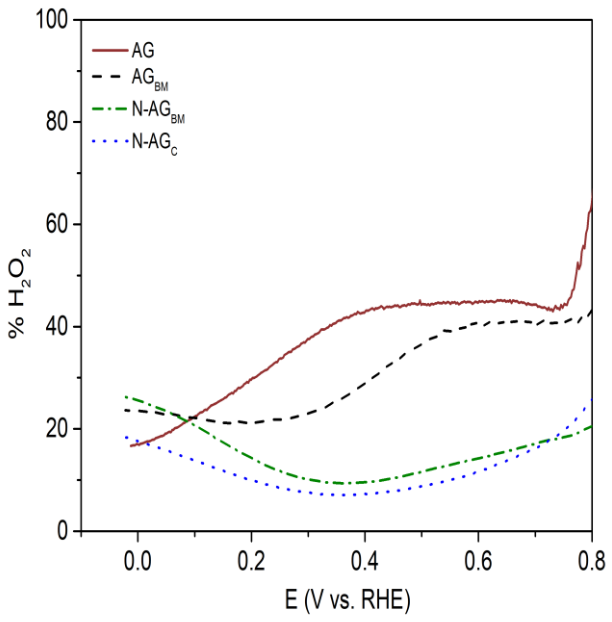

Figure S13: Hydrogen peroxide production for activated samples.
